# An Ulcerated Ileal Gastrointestinal Stromal Tumor Disguised as Acute Appendicitis

**DOI:** 10.1155/2018/1320107

**Published:** 2018-06-05

**Authors:** Ashish Lal Shrestha, Girishma Shrestha

**Affiliations:** ^1^Department of General Surgery, United Mission Hospital, Tansen, Palpa, Nepal; ^2^Department of Pathology, Patan Academy of Health Sciences, Lagankhel, Kathmandu, Nepal

## Abstract

**Background:**

Gastrointestinal stromal tumor (GIST) of the ileum is not a common differential to consider in the management of acute right iliac fossa (RIF) pain and tenderness. Finding of a normal-looking appendix intraoperatively should arouse the surgeon to explore further and look for other unanticipated pathologies. We present a case, clinically diagnosed as acute appendicitis and intraoperatively found to be an ulcerated ileal GIST.

**Case Presentation:**

A 28-year-old female without previous comorbidities presented to the emergency unit with sudden pain around the umbilicus that later migrated and localized to the RIF for one day. There was associated intermittent fever and anorexia without urinary symptoms. Abdominal examination revealed guarding and rebound tenderness at RIF. Examination by 2 senior surgeons at different points of time, the same day, made a clinical diagnosis of acute appendicitis. Ultrasonogram (USG) was inconclusive. At laparotomy through Lanz incision, the appendix was found to be normal and no other pathology was identified on walking bowel up to 3 ft proximal to ileocecal junction (ICJ). Just when closure was thought of, an ulcerated lesion could be seen through the medial aspect of the incision. On further exploration, a 7 × 5 cm ulcerated lesion arising from the antimesenteric border of the ileum was noted with localized interloop hemoperitoneum and inflammatory exudates. Ileal segmental resection anastomosis was done with peritoneal toileting. The lesion was subsequently reported to be an ulcerated malignant GIST.

**Conclusion:**

The commonest cause of RIF pain with localized peritonitis is an acutely inflamed appendix. Dilemma arises when the appendix is found to look normal. Further exploration is indicted to not miss other findings.

## 1. Introduction

The term “GIST” was first introduced by Mazur and Clark in 1983 to include the nonepithelial tumors of digestive tract that lack ultrastructure of smooth muscle cells and immunohistochemical properties of Schwann cells. GISTs are known to arise from the interstitial cells of Cajal that are regarded as the pacemaker cells, constituting a part of the autonomic nervous system of the gut and controlling intestinal peristalsis [1]. GISTs may vary in presentation and sometimes mimic other commoner conditions. We report an interesting case of an ulcerated small bowel GIST that behaved clinically like acute appendicitis. The clinical presentation, investigative findings, and management are discussed along with relevant literatures.

## 2. Case Presentation

**Figure 1:**
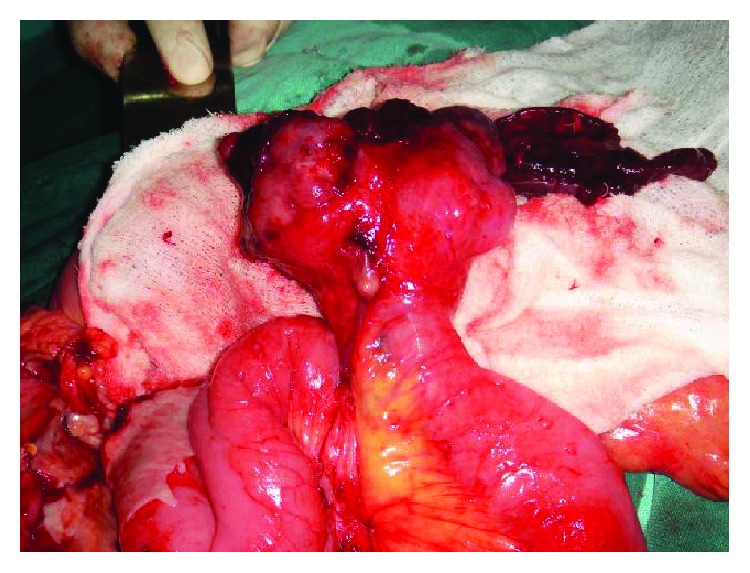
The intraoperative image of the ulcerated ileal GIST arising from the antimesenteric border with interloop hemoperitoneum and inflammatory exudates.

A 28-year-old female with insignificant past medico surgical history presented with one day of acute onset pain in the periumbilical region that later migrated and confined to the RIF. She had associated intermittent fever, nausea, and loss of appetite. She did not have any urinary symptoms, bowel irregularities, or gynecological complaints. Abdominal examination was performed by two senior surgeons at two different occasions; the same day had findings of guarding and rebound tenderness at RIF. Hematological tests showed polymorphonuclear leukocytosis with left shift. Biochemical tests and urinalysis were normal. Urinary pregnancy test was negative. Abdominal radiographs were unremarkable. USG could not visualize appendix and was inconclusive except for probe tenderness in RIF. CT scan of the abdomen could not be done due to unavailability. A clinical diagnosis of acute appendicitis was made assigning an Alvarado score of 9/10. Laparotomy was performed using the Lanz incision in RIF. Intraoperatively appendix was found to be normal without evidence of inflammation or infection in RIF. In view of symptoms and signs, a possibility of other pathology was thought. Walking the bowel proximally up to 3 feet (1 m) did not show a Meckel's diverticulum or any other small bowel lesions. There were no obvious mesenteric lymph nodal enlargement and pelvic organs looked pristine. Approaching closure, just when the medial edge of the incision was retracted superomedially, a hemorrhagic lesion seemed to appear little deeper in the mid abdomen. Therefore, the incision was extended transversely from the medial edge to explore further. Entire bowel was explored and this revealed an ulcerated lesion measuring 7 × 5 cm arising from the antimesenteric border of the ileum 8 feet (2.5 m) from ICJ with localized interloop hemoperitoneum and inflammatory exudates as shown in Figure 1. Resection of ileal segment containing the lesion was performed followed by restoration of bowel continuity and peritoneal toileting. The lesion was subsequently reported to be an ulcerated malignant ileal GIST.

Histopathologically, gross examination confirmed the operative findings, and the cut section revealed a nodular lesion protruding out of the serosal surface measuring 7 × 5 cm along with 2 lymph nodes each measuring 2 × 1 cm.

**Figure 2:**
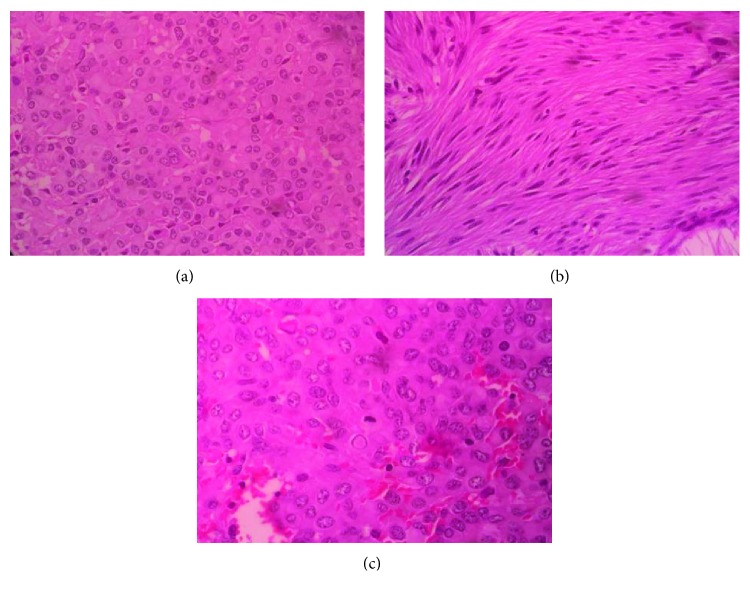
Microscopic appearance of the ileal malignant GIST (stained with eosin/hematoxylin stain) under high-power magnification showing mitotic figures. (a) Tumor cells exhibiting epithelioid morphology (H&E; 20x). (b) Spindle cells in fascicles (H&E; 20x). (c) Areas with frequent mitotic activity (H&E; 40x).

Microscopically, the growth from the ileum had villous lining epithelium with focal ulceration. The submucosal region had a circumscribed nodule with proliferation of loosely cohesive spindle cells; some of which were arranged in vague storiform pattern and others in long fascicles. There were areas with epitheloid cells forming small anastomosing nests and cords. The areas in between these showed skenoid fibers along with focal areas of hemorrhage, infarction, and congestion as shown in Figure 2. The mitotic figures were seen (8/50 high-power field). The lymph nodes were microscopically identified to be reactive, and the resected margins of the ileum were free of tumor.

Based on tumor size and mitotic activity, possibility of a malignant GIST was suggested along with immunohistochemical analysis (CD117 and CD34) for further confirmation. The patient had an uneventful recovery and was discharged on the 8th postoperative day. She was advised to review a week later at the outpatients but failed to report. All possible contacts were used to trace her, but she remained inaccessible and lost to follow-up.

## 3. Discussion

**Figure 3:**
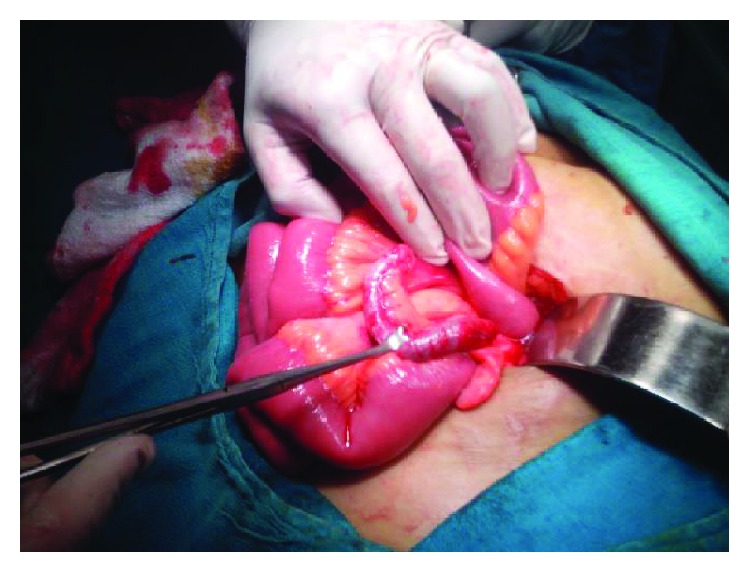
An uncomplicated appendicitis in a different patient.

It is a common clinical situation to have a patient presenting with periumbilical pain subsequently localizing to the RIF associated with vomiting with or without nausea and fever. The classical symptom complex called Murphy's triad is often observed and tends to occur in the same sequential order [2]. The findings of guarding at the RIF with McBurney's point tenderness are suspicious of acute appendicitis along with various named signs [3]. Leukocytosis and neutrophilic left shift added to the USG findings of a noncompressible blind tube > 6 mm in RIF with probe tenderness strongly impress upon the surgeon to wait no further before embarking on an emergency appendectomy. The usual finding is that of an inflamed appendix with or without associated complications (gangrene, perforation, or periappendicular collection).  Figure 3 shows an uncomplicated appendicitis in a different patient.

The annual global incidence of appendicitis is reported to be 11 cases per 10,000 population [4]. In one study, the sensitivity and specificity of clinical examination to diagnose appendicitis were 99% and 76% and the same for USG were 99% and 91%, respectively [5]. Various scoring systems have also been devised to aid accurate preoperative diagnosis, for example, Alvarado, Ohhmann, Eskelinen, and RIPASA, and report a wide range of variability in sensitivity, specificity, and predictive validity in different comparative studies [6]. Despite our long-term experience in treating this condition, there have been several incidences of finding an unanticipated pathology intraoperatively and the “On Table Surprise” does not stop to amaze us even now. In most series, a negative appendectomy rate of 10–20% is considered acceptable though newer studies quote an even lesser rate [7, 8]. A normal-looking appendix certainly arouses the surgeon to suspect something sinister and thence the usual tendency to look for conditions like an inflamed Meckel's diverticulum, the incidence of which is said to be 2%. The other pathologies that may be encountered are mesenteric lymphadenitis, large or small bowel diverticulitis, right ureteric pathology, and a wide variety of gynecological ailments like ruptured ovarian follicle with midcycle ovulatory bleeding (Mittelschmerz's), ovarian torsion, salpingitis, and ruptured ectopic pregnancy especially in women of child bearing age [9]. But a ruptured small bowel GIST is certainly not the prime suspect under usual circumstances. GISTs are known to us since the time they were first reported by Mazur and Clark in 1983. They have constantly made their presence felt in various case reports globally when they were not recognized preoperatively. Refractory peptic ulcer disease, gastrointestinal bleeding, pneumomediastinum, acute diffuse peritonitis, abdominal abscesses, and sudden perforation with hemoperitoneum have all been the various modes of presentation of GISTs [10–15]. One similar incidence of a GIST mimicking appendicitis was found reportedly from the jejunum; however, ours was one from the ileum [16]. The usual age of presentation of GIST is 40 to 60 years and the common sites of origin are the stomach and followed by small bowel and colorectum and rarely esophagus. Although many are diagnosed incidentally, some with advanced disease present with symptoms that include nonspecific abdominal pain and large abdominal masses. Occasionally, luminal erosion of a highly vascular GIST may present with a life-threatening gastrointestinal hemorrhage, while on account of luminal narrowing, the other forms of presentation may be obstruction and perforation. Tumor rupture in this regard seems to be more dreadful condition; in that, it carries risks of tumor dissemination that can be difficult to treat apart from hemoperitoneum and acute abdomen. Some may even present with metastasis to the liver and peritoneum and very rarely to the lungs. Also of note are local spread to adjacent viscera like the intestine, omentum, and diaphragm. Cross-sectional imaging with a CT scan is helpful in identifying the extent of lesion and studying the characters like necrosis, ulceration, calcification, ascites, and local and distant metastasis that denote the aggressive nature of primary lesion to plan a subsequent operative therapy. PET scan is considered an important adjunct to CT in evaluation and in order to assess response to chemotherapy [1]. In an acute setting, and in a peripheral set up like ours, both these modalities are only of theoretical value. Similarly, endoscopic ultrasound and FNAC are invaluable in preoperative tissue diagnosis in centers where the expertise is available. Definitive diagnosis is possible with histopathological examination of the tissue aided by immunohistochemistry (IHC); the current panel of which includes CD117, smooth muscle actin, CD34, desmin, and S-100. Unfortunately, in our case, it was unavailable and had to be sent to a tertiary care center on receiving the histopathological report. Since the patient did not follow up in the postoperative period, it could not be done. An extensive search in PubMed, Medline, and Google in reference to GIST misdiagnosed as appendicitis was done from 2000 till now. Only 4 cases were found to have been reported worldwide of which 1 was in the stomach, 2 were in the jejunum, and 1 was in the ileum [16–19]. This was the second report of similar presentation of GIST in the ileum. In all the cases, treatment approach was surgical with laparoscopic resection in 1 and open resection in the rest. Our patient underwent open resection with an uneventful recovery. Had she returned for follow-up, she should have been evaluated for metastatic disease and further management. But since that was not possible, we could neither plan further treatment nor prognosticate her disease. In general, prognosis of GIST depends upon the size of the tumor and to the mitotic rate: tumors > 10 cm or with a mitotic rate of >5 per 50 HPF having higher risk of recurrence, metastatic spread, and a poorer prognosis. Other prognostic factors include tumor-free surgical margins, tumor rupture, and c-kit mutation [17]. The IHC and other molecular studies could not be done in our patient.

## 4. Conclusion

In essence, an ulcerated malignant GIST of the ileum masquerading as acute appendicitis is a common presentation of an uncommon diagnosis. Disproportionate symptoms and signs inconsistent with a normal-looking appendix on table should alert the surgeon to suspect other possible causes no matter how remote. Negative appendectomy should not be taken lightly and mandates thorough exploration of the entire length of bowel. Definitive diagnosis is possible on histopathological evaluation aided by IHC. Resection with negative margins and further therapy based on IHC panel forms the backbone of management. The awareness of the clinical presentation and good pathological expertise are important adjuncts in the diagnosis. Surgery is the mainstay of treatment in the acute presentation.

## Abbreviations

GIST: Gastrointestinal stromal tumor
RIF: Right iliac fossa
USG: Ultrasonogram
ICJ: Ileocecal junction
CT: Computed tomography
IHC: Immunohistochemistry.
